# Symphyseal fixation in open book injuries cannot fully compensate anterior SI joint injury—A biomechanical study in a two-leg alternating load model

**DOI:** 10.1371/journal.pone.0184000

**Published:** 2017-11-27

**Authors:** Fabian M. Stuby, Mark Lenz, Stefan Doebele, Yash Agarwal, Hristo Skulev, Björn G. Ochs, Jörn Zwingmann, Boyko Gueorguiev

**Affiliations:** 1 BG Trauma Center, Eberhard Karls University, Tuebingen, Germany; 2 Department of Trauma, Hand and Reconstructive Surgery, University Hospital Jena, Jena, Germany; 3 AO Research Institute Davos, Davos, Switzerland; 4 Department of Materials Science and Technology, Technical University Varna, Varna, Bulgaria; 5 Department of Orthopaedic and Trauma Surgery, University of Freiburg Medical Center, Freiburg, Germany; Universite de Nantes, FRANCE

## Abstract

**Introduction:**

In open book injuries type Tile B1.1 or B1.2 also classified as APC II (anteroposterior compression), it remains controversial, if a fixation of the anterior ring provides sufficient stability or a fixation of the posterior ring should be included. Therefore the relative motion at the sacroiliac joint was quantified in a two-leg alternating load biomechanical pelvis model in the intact, the injured and the restored pelvis.

**Methods:**

Fresh-frozen intact (I) pelvises (n = 6) were subjected to a non-destructive cyclic test under sinosuidal axial two-leg alternating load with progressively increasing amplitude. Afterwards an open book injury (J) including the anterior ligament complex of the left sacroiliac joint, the sacrospinal and sacrotuberal ligaments (Tile B1.1) was created and the specimens were retested. Finally, the symphysis was stabilized with a modular fixation system (1-, 2- or 4-rod configuration) (R) and specimens were cyclically retested. Relative motion at the sacroiliac joint was captured at both sacroiliac joints by motion tracking system at two load levels of 170 N and 340 N during all tests.

**Results:**

Relative sacroiliac joint movements at both load levels were significantly higher in the J-state compared to the I-state, excluding superoinferior translational movement. With exception of the anteroposterior translational movement at 340N, the relative sacroiliac joint movements after each of the three reconstructions (1-, 2-, 4-rod fixation) were significantly smaller compared to the J-state and did not differ significantly to the I-state, but stayed above the values of the latter. Relative movements did not differ significantly in a direct comparison between the 1-rod, 2-rod and 4-rod fixations.

**Conclusion:**

Symphyseal locked plating significantly reduces relative movement of the sacroiliac joint in open book injuries type Tile B1.1 or B1.2 (APC II) but cannot fully restore the situation of the intact sacroiliac joint.

## Introduction

External rotation injury of the pelvic ring, the so called open-book injury needs accurate reduction and stable fixation, at least if the posterior arch is involved [1–4]. The Young-Burgess classification system categorizes this type of injury as anteroposterior compression (APC) injury [1, 5], in the Tile classification, which is commonly used in Europe it is named B1.1 or B.1.2. The type B1.1 is characterized by a purely ligamentous injury of the anterior iliosacral ligaments, the B1.2 injury includes an anterior fracture of the lateral sacral bone at the iliosacral ligaments insertion. Depending on the amount of symphysis’ diastasis and the involvement of only the anterior ligament complex or both the anterior and posterior complex of the sacroiliac joint, it is further subdivided into three groups. In Tile B1.1 injuries, apart from the symphysis only the anterior sacroiliac ligament complex is injured, but the posterior sacroiliac ligament complex remains intact. This type of injury frequently occurs in motorcycle accidents, fall from height and equestrian [2]. It is also discussed to which degree of symphyseal opening a tear of the sacratuberous and sacraspinos ligaments occur and how they influence stability of the pelvic ring and of its fixation. As we only produced a 2.5 cm diastasis of the symphysis and an anterior injury of the iliosacral ligaments, we might have produced a limited instability compared to that of a large diastasis occurring in accidents of living humans [6–9]. Various fixation methods have been described for traumatic pubic symphysis disruptions. The anterior ring, i.e. the symphysis is stabilized mainly by plate fixation. The posterior ring is fixed predominantly by iliosacral screws or transiliosacral bars [4, 10]. The extent of stabilization depends on the degree of instability. In open book injuries type Tile B1.1, it remains controversial, if a fixation of the anterior ring alone provides sufficient stability or if a fixation of the posterior ring should be included [1, 2]. Theoretically, in absence of vertical and rotational instability due to intact posterior sacroiliac ligaments, an anterior stabilization, closing the pelvic ring should suffice. Recent follow-up studies revealed that a remarkable amount of patients had a premature postoperative fixation failure [2] and that additional stabilization of the posterior ring decreases anterior plate failure and malunion rate [1]. To quantify the relative motion at the sacroiliac joint, acquired data of a two-leg alternating load biomechanical pelvis model [11] are used. Relative motion at the sacroiliac joint in the intact pelvis, the injured (Tile B1.1) pelvis and the restored pelvis (anterior plate) are evaluated.

As we only produced a ligamentous injury we only refer to Tile B1.1 and not to Tile B1.2. The biomechanical setup was established to evaluate movements of the complete pelvic ring in different stability modes. For the posterior part we hypothesized that restoration of the pelvic ring by an anterior plate may not fully reestablish the stability of the intact pelvis.

## Materials and methods

This investigation was approved by the institutional internal review board, based on the approval of specimens' delivery by Science Care Ethics Committee. All donors have given a signed agreement for scientific medical research and education during their lifetime. None of the donors were from a vulnerable population and all donors or next of kin provided written informed consent that was freely given.

Additional non-published data from a previously published test [12] concerning the relative motion at the sacroiliac joint were separately evaluated. Therefore, the study design is identical to this previous work.

### Specimens

Six human pelvises including proximal femora and vertebra L5 with no evidence of bone and soft tissue pathology were harvested from fresh-frozen (−20°C) cadavers of 3 male and 3 female donors, mean age 75 years, mean body height 167 cm and mean body weight 71.5 kg. Donors with diseases or medical history, that might have influenced bony and ligaments structures, have been excluded. Radiological imaging was performed to exclude defects affecting the integrity of the pelvic structure. Specimens were thawed 48 hours at room temperature prior to preparation and biomechanical testing. Ligaments integrity was checked by hand. The soft tissue was removed preserving the pubic symphysis, sacroiliac ligaments, iliolumbar ligaments, proximal femoral ligaments and hip joints with their capsulae. The L5 vertebra was separated and used for assessment of bone mineral density (BMD) applying high-resolution peripheral quantitative computed tomography (HR-pQCT, XtremeCT, Scanco Medical, Brüttisellen, Switzerland) with a resolution of 123 μm and a volume of interest defined as a cylinder with a length of 6 mm and a diameter of 15 mm in the vertebral body. The proximal femora were sectioned at a distance of 200 mm from the lesser trochanter. The sacrum was equipped with a 10 mm petroleum jelly-coated stainless steel rod, passing through a hole drilled from the base of the sacrum to the S3–S4 region. Subsequently, the sacrum was embedded in polymethylmethacrylate (PMMA; Beracryl,W. Troller Kunststoffe AG, Switzerland) which reinforced the bone to hold sufficient load during biomechanical testing. Finally, five clusters of four infrared light-reflecting markers each were attached to either side of the pubic symphysis, the sacrum and the left and right iliac crests of each specimen for three-dimensional (3D) motion tracking.

### Injury

The open book injury was produced by scalpel dissection of the pubic symphysis, the left anterior sacroiliac joint, and the sacrospinal and sacrotuberal ligaments on the left side. Subsequently, the pelvic ring was opened until a gap of at least 3 cm was measured at the symphysis. This produced an instability similar to an open book injury (Tile B1.1 or APC II according to Young Burgess).

### Implant

To achieve a stabilization of the ruptured symphysis with different modes of flexibility, a special modular implant device was constructed. The aim was to equip the specimen with a single base plate that could accommodate 1-, 2- or 4-rod configurations. The modular implant device was designed such that its bending stiffness in the 3-rod configuration resembled the bending stiffness of a standard symphyseal locking plate (3.5 mm 4-hole LCP DePuy Synthes, Zuchwil, Switzerland) made from stainless steel 316L. Prior to the biomechanical tests of the different flexible fixation methods each specimen underwent biomechanical testing in the intact and injured condition to gain baseline values for further comparison as described below [11].

### Instrumentation

The baseplate of the stabilization device was fixed to either side of the symphysis using two 3.5 mm locking screws with 50 mm length before simulating the injury. As the instrumentation was performed in intact condition and therefore with physiological distances of the symphysis structures we did not use compression for the reassembling after injury. Stabilization of the dorsal part of the pelvic ring was not performed. Each pelvis was tested in the intact (I), the injured (J) and the respective reconstructed (R) states. The order of the 1-2-4-rod instrumentations was randomized according to a previously published study [12].

### Biomechanical testing, test setup and loading protocol

Biomechanical testing was performed on a biaxial servo-hydraulic testing machine MTS Mini Bionix II 858 (MTS Systems Corp., USA) with 25 kN/250 Nm load cell. The setup for biomechanical testing was adopted from a previous study where two-leg alternate loading was introduced to investigate fixation methods of the pelvic ring with focus on the pubic symphysis [11].

**Fig 1:**
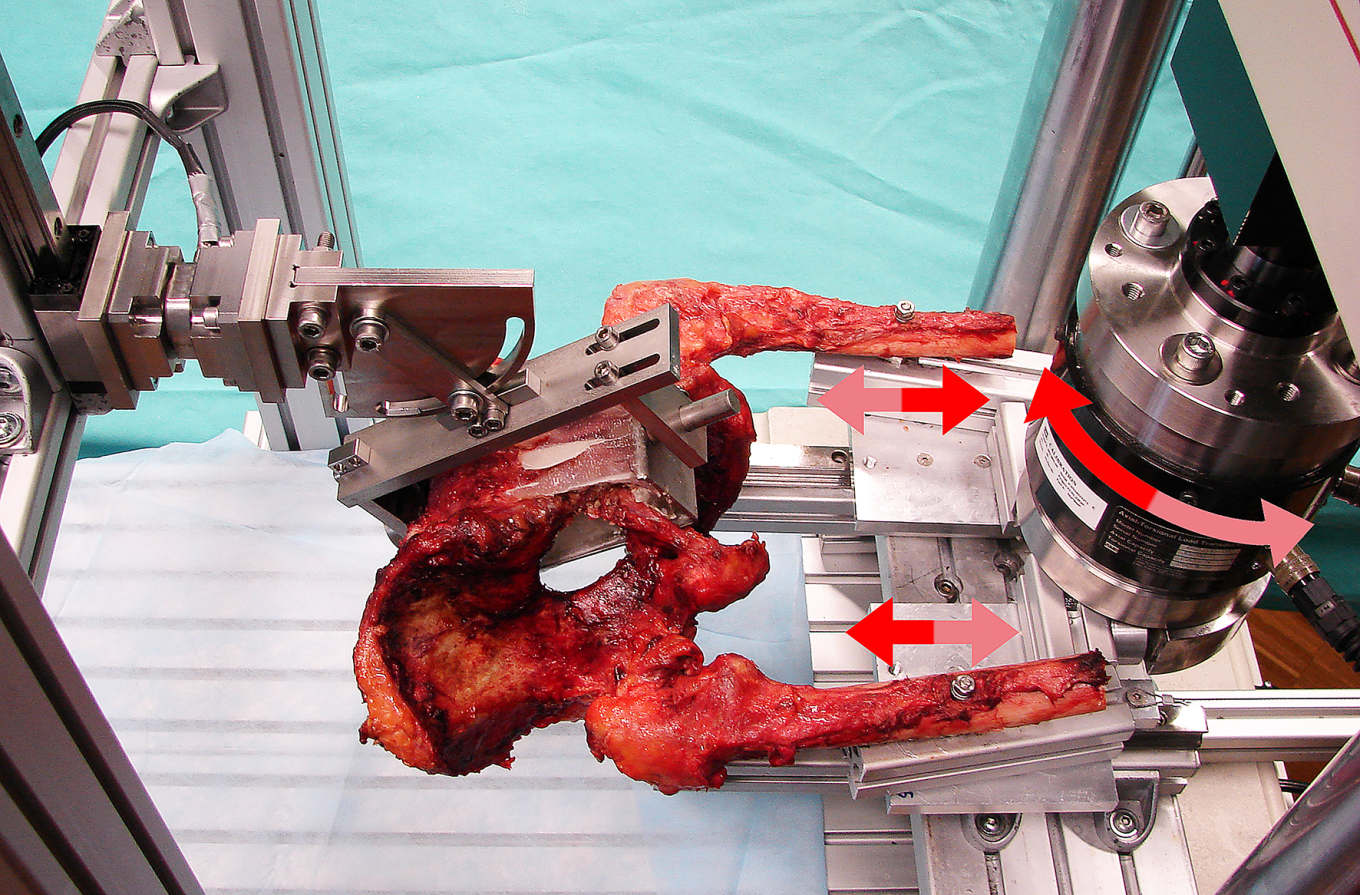
Test setup with a pelvic specimen mounted horizontally in the machine frame for biomechanical testing (posterolateral view). Both distal femoral ends are attached to sliding posts and alternately loaded in the direction of their mechanical axes (as indicated with the two straight double arrows) via converted torsional movement of the machine actuator with the load cell (as indicated with a curved double arrow). The sacrum is attached to the testing frame via a jig allowing free movement in the sagittal plane.

Each pelvis was mounted horizontally in the test frame as shown in Fig 1. The proximal femora were placed in such a way that their shafts formed an angle of 9° with the symmetry axis in the coronal plane. Both distal femoral ends were attached to sliding posts using bolts. Each femur was loaded or unloaded in direction of its mechanical axis (running from the center of the femoral head to the intercondylar notch) via torsional movement of the machine actuator. Applied torque was proportional to the physiological load on each femur during walking. The jig for sacral fixation was mounted on a Kistler load-cell, which was free to move in the sagittal plane. The load-cell allowed monitoring and measurement of the vertical and horizontal loads at its attachment point in order to ensure symmetric loading on both legs. A non-destructive cyclic test was run at 1 Hz. Whereas the valley load of each femur was kept at a constant level of 0 N, the peak load was progressively increasing from 170 N to 340 N over 1000 cycles at a rate of 0.17 N/cycle. This loading was found to be within the safe limits, such that no instability or disruption would be expected [9, 13].

### Data acquisition and analysis

**Fig 2:**
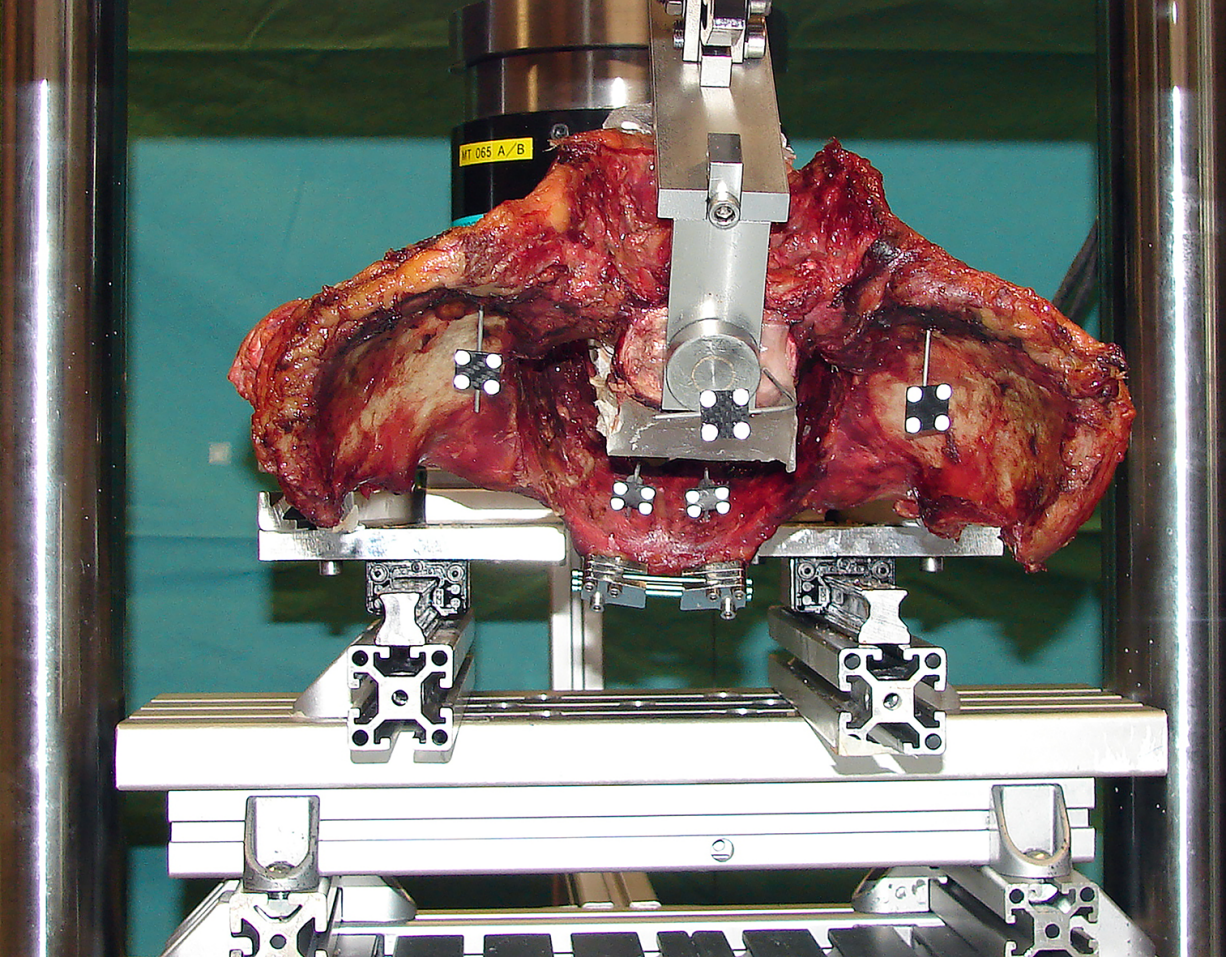
Axial view of a pelvic specimen with five marker sets attached on its sacrum, iliac and pubic bones for motion tracking. The specimen is mounted on the testing frame with a jig holding the sacrum. The pubic symphysis is fixed with a modular implant device.

The axial load was continuously recorded from the testing machine controllers at 128 Hz. Inter-segmental movements were registered optically in all six degrees of freedom with 3D motion tracking, monitoring the 5 retro-reflective marker clusters mounted on each specimen with five infrared digital cameras (ProReflexMCU, Qualisys AB, Gothenburg, Sweden) at a rate of 100 Hz (Fig 2). The co-ordinate system used for the sacroiliac joint was adopted from previous publications [14–18]. Its center was located at the most superior aspect of the joint and the frontal, transverse and sagittal planes were defined as XY, XZ and YZ, respectively. The axes perpendicular to these planes were called Z (sagittal horizontal axis), Y (vertical axis) and X (frontal horizontal axis), respectively. Rotational and translational motion components at the most superior aspect of the sacroiliac joint were first calculated in all six degrees of freedom from the optical motion tracking data using a script developed in Matlab software package (MathWorks, Natick, MA, USA). Based on this, a parameter of interest called ‘relative movement’ was defined for each component separately as its peak-to-valley amplitude over one cycle (between loaded and unloaded state) at the beginning and the end of the non-destructive cyclic test (at peak load of 170 N and 340 N, respectively) to characterize the elastic joint movements during those specific cycles.

The values of the parameters of interest were selected for statistical analysis which was performed using SPSS software package (IBM, Chicago, IL, USA). Normal distribution of all data was screened with the Shapiro–Wilk Test. Statistical analysis of the different stabilization methods was performed with the General Linear Model Repeated Measures Test. Level of significance was set at P = 0.05 for all statistical tests.

### Ethics statement

The study was reviewed and approved by the institutional internal review board of the AO Research Institute Davos /Switzerland, based on the approval of the specimens' delivery by Science Care Ethics Committee.

All donors have given a signed agreement for scientific medical research and education during their lifetime.

None of the donors were from a vulnerable population and all donors or next of kin provided written informed consent that was freely given.

## Results

The BMD of the specimens, as measured in L5, was 65 ± 18.6 mgHA/ccm (mean ± standard deviation, S1 Appendix), which was within the range of the average population [11, 19]. Relative movements in all directions of left versus right sacroiliac joint in the intact state did not differ significantly (p>0.05).

The relative movement in rotation and translation of the left sacroiliac joint in all 5 biomechanical tests (intact, injured, 1-rod fixation, 2-rod fixation, 4-rod fixation) together with the p-values for the respective comparisons are given in Table 1.

**Table 1:** 

LEFT SI joint (injured)													
Rot [deg] Tr [mm]	Load [N]	Mean±SD (standard deviation)					P-values						
		I (int)	J (inj)	1-rod (1-R)	2-rod (2-R)	4-rod (4-R)	I vs J	1-R vs I	1-R vs J	2-R vs I	2-R vs J	4-R vs I	4-R vs J
**Rx**	**170**	0.76 ±0.27	2.04 ±1.18	1.22 ±0.88	1.17 ±0.83	1.14 ±081	**0.03**	0.29	**0.04**	0.35	**0.04**	0.55	**0.03**
	**340**	1.65 ±0.71	4.66 ±2.42	2.64 ±0.98	2.6 2±1.08	2.54 ±1.05	**0.02**	0.21	**0.04**	0.26	**0.04**	0.37	**0.03**
**Ry**	**170**	0.51 ±0.32	1.76 ±1.35	0.66 ±0.47	0.63 ±0.42	0.61 ±0.39	**0.03**	0.48	**0.04**	0.60	**0.04**	0.87	**0.04**
	**340**	0.85 ±0.44	3.62 ±2.87	0.92 ±0.47	0.87 ±0.46	0.84 ±0.44	**0.03**	0.38	**0.04**	0.56	**0.04**	0.63	**0.04**
**Rz**	**170**	0.68 ±0.32	1.61 ±0.56	0.91 ±0.51	0.89 ±0.42	0.85 ±0.37	**0.03**	0.15	**0.04**	0.17	**0.04**	0.28	**0.03**
	**340**	1.23 ±0.49	3.50 ±1.74	2.40 ±0.93	2.33 ±0.92	2.28 ±0.78	**0.02**	0.11	**0.04**	0.15	**0.03**	0.22	**0.03**
**Tx**	**170**	0.36 ±0.17	1.30 ±0.91	0.5 2±0.44	0.49 ±0.39	0.47 ±0.32	**0.03**	0.43	**0.04**	0.73	**0.03**	0.79	**0.03**
	**340**	0.87 ±0.51	2.17 ±0.98	1.04 ±0.69	0.99 ±0.64	0.96 ±0.61	**0.02**	0.25	**0.03**	0.32	**0.02**	0.54	**0.02**
**Ty**	**170**	0.40 ±0.34	0.48 ±0.17	0.46 ±0.22	0.44 ±0.24	0.43 ±0.27	0.42	0.13	0.68	0.25	0.54	0.35	0.49
	**340**	0.75 ±0.37	0.94 ±0.64	0.92 ±0.47	0.89 ±0.42	0.88 ±0.39	0.26	0.11	0.35	0.20	0.33	0.23	0.29
**Tz**	**170**	0.61 ±0.23	1.73 ±1.18	1.28 ±0.91	1.22 ±0.86	1.16 ±0.83	**0.03**	0.12	**0.04**	0.17	**0.04**	0.19	**0.03**
	**340**	1.18 ±0.37	3.54 ±2.05	2.33 ±1.13	2.28 ±1.10	2.23 ±1.03	**0.02**	**0.03**	**0.04**	**0.03**	**0.03**	**0.03**	**0.03**

Left: Rotational (R) and translational (T) iliosacral joint movements for the intact (I), injured (J) and reconstructed (R) specimen states, with 1-rod, 2-rod and 4-rod implant configurations, in terms of mean and standard deviation (SD) at 170N and 340N load. Right: P-values for the respective comparisons of the rotational and translational iliosacral joint movements between the intact, injured and reconstructed states. Significant p-values identified bold.

All relative movements in rotation and translation increased significantly between 170N and 340N load for each specimen's state (intact, injured, 1-rod fixation, 2-rod fixation, 4-rod fixation) during the respective test (p<0.05).

All relative movements at 170 and 340 N loading were significantly higher in the injured non-reconstructed J-state compared to the intact I-state, excluding the superoinferior translational movement which did not differ significantly between the intact and injured state.

With exception of the anteroposterior translational movement at 340N, the relative sacroiliac joint movements after each of the three reconstructions (1-rod fixation, 2-rod fixation, 4-rod fixation) were significantly smaller compared to the injured J-stated and did not differ significantly to the intact I-state, but remained above the values of the latter.

The relative movements in all directions did not differ significantly in a direct comparison between the 1-rod, 2-rod and 4-rod fixations with p>0.05 in all statistical tests.

## Discussion

Relative movements at the sacroiliac joint were quantified in a two-leg alternating load biomechanical pelvis model in the intact state, after Tile B1.1 (APC II) injury and after anterior plate reconstruction. This model is capable to simulate the effect of the shear forces that are caused by the alternating load during walking, in contrast to previously published models for biomechanical testing of the pelvic ring with either simultaneous two-leg stance loading [4, 20–22] with simultaneous load application on both hips via the spine, or one-leg stance loading [13, 23–25], where the force is applied to the pelvic ring via a single femur and the iliac crest is balanced with wires tied around the crest providing muscle force compensation. Tile B1.1 injury revealed a significant increase in sacroiliac joint relative movements in all directions except superoinferior translation compared to the intact state of the ipsilateral side. As already postulated by the current classification systems of Tile and Young-Burgess [3, 5, 26], the open-book injury type is not indicated with posterior vertical instability. This situation was reproduced very well in our model where the translational movements at the iliosacral joint along the vertical axis were comparable between the intact and injured states. Therefore, in absence of obvious posterior instability, anterior plating without posterior ring stabilization is usually performed [2]. In line with our previous results [12], Abdelfattah et al. observed a significantly higher vertical relative movements at the pubic symphysis after anterior sacroiliac ligament dissection [27]. The sacrospinous and sacrotuberal ligaments contribute mainly to rotational but moreover to vertical stabilization of the sacrum [28].

A magnetic resonance imaging study on Tile B1.1 (APC II) injuries revealed that the posterior sacroiliac ligaments remained intact [1, 29]. Simulations of a Tile B1.1 injury in a single leg stance model show that anterior pubic plate fixation of the pelvic ring does not sufficiently stabilize the sacroiliac joint compared to the intact state [13]. Multiple fixation strategies based on biomechanical studies have been proposed [30–32]. Clinically, the use of multihole plates compared to the formerly used two-hole plates decreased the rate of anterior plate failure and malunion [1, 33]. Locked symphyseal plating systems initially provide better stability than non-locked systems for anterior-posterior shear translation of pelvic ring fractures [34] but also seem to show earlier loosening [12]. Our results revealed that reconstruction of the anterior ring using a four-hole locking screw fixation (two-screws per side) exhibited a significant reduction of relative motion at the sacroiliac joint compared to the injured state but does not fully restore the intact state. Especially in the anteroposterior translation, a significant difference in-between the reconstructed and the intact state remained at higher load.

Varga et al. pointed out that the inferior part of the symphysis is loaded in tension and is consecutively not adequately reconstructed by a superior plate [4], effecting higher relative movements at the sacroiliac joint. Apart from its tensional loading, the inferior part of the symphysis stabilizes the pelvic ring against vertical translation and internal rotation [28]. Two publications by Avilucea et al. and Simonean et al. showed that iliosacral screw or iliosacral plate fixation reduces the relative movement at the sacroiliac joint significantly in Tile B1.1 and B1.2 (APC II) injuries, whereas the type of anterior fixation has a minor influence on sacroiliac joint stability [1, 35]. Our results indicate that a single locking plate does not adequately stabilize the inferior part of the symphysis, since higher relative movements at the sacroiliac joint persist after anterior reconstruction. Stabilization of the inferior part of the pubic symphysis by anterior double plating increases the stability and significantly reduces the relative movement in the sacroiliac joint compared to single superior plating [4]. Although we did not observe a significant difference of relative movements in-between the one to four rod fixation of the symphysis, it has been visualized [28] that a sufficient stability of the symphyseal ligaments reduces the load at the posterior pelvic ring.

Our study has some limitations as follows. The open book injury of the pelvis is an injury of the younger population and commonly the result of a high impact trauma. The available specimens for this biomechanical study had an average age of 75 years and therefor did not fully satisfied the anatomical requirements for bony and ligamentous structures which we wished to have. Although we did not consider muscle forces, additionally stabilizing and distracting the pelvis, we developed a two leg alternating stance model for this test to simulate pelvic movement during walking and to maximize alternating load and translational shear load. The sacrum was fixed in the central portion. This fixation was necessary because of lacking muscle forces. In addition, the randomized cyclic test sequences with different implant configurations were performed under relatively low axial loads of up to 340 N, which were smaller than the forces acting in vivo. Such test design aimed to guarantee the non-destructive fashion of testing. Nevertheless, some wear still occurred of the specimens' ligaments during each test as a consequence of the loading. Since data were additionally acquired during a study on pubic symphysis stabilization, we did not investigate the influence of additional iliosacral screw fixation or symphyseal double plating on the relative movements at the sacroiliac joint, so that our results have to be compared to the existing literature.

To exclude an influence of an unavoidable imbalance (non-symmetry) of the left and right sacroiliac joints on the test results, relative movements of the different states (intact, injured, reconstructed) were only compared within the ipsilateral side and not to the contralateral one. Although there was some difference in the direct comparison of the left versus right sacroiliac joint of each specimen, this could not reveal a statistical significance and is therefore not further commented.

## Conclusion

Symphyseal plating significantly reduces relative movement of the sacroiliac joint in open book injuries type Tile B1.1 or B1.2 (APC II) but cannot fully restore the stability of the intact sacroiliac joint. This may explain to some extent the implant failure and lack of healing at the sacroiliac joint seen in this type of injury if only the pubic symphysis is stabilized without additional sacroiliac joint stabilization.

## Supporting information

S1 Appendix Individual values for BMD and relative movements at the left sacroiliac joint of each specimen. Rotational and translational movements are presented for all biomechanical tests with intact, injured, 1-rod fixation, 2-rod fixation and 4-rod fixation specimen's state under 170N and 340N load.
(XLSX)

## References

[1] Avilucea FR, Whiting PS, Mir H. Posterior Fixation of APC-2 Pelvic Ring Injuries Decreases Rates of Anterior Plate Failure and Malunion. The Journal of bone and joint surgery American volume. 2016;98(11):944–51. Epub 2016/06/03. doi: 10.2106/JBJS.15.00723 . 27252439 10.2106/JBJS.15.00723

[2] Eastman JG, Krieg JC, Routt ML Jr. Early failure of symphysis pubis plating. Injury. 2016;47(8):1707–12. Epub 2016/06/11. doi: 10.1016/j.injury.2016.05.019 . 27282685 10.1016/j.injury.2016.05.019

[3] Tile M, Helfet D, Kellam J. Fractures of the Pelvis and Acetabulum: Lippincott Williams & Wilkins; 2003.

[4] Varga E, Hearn T, Powell J, Tile M. Effects of method of internal fixation of symphyseal disruptions on stability of the pelvic ring. Injury. 1995;26(2):75–80. Epub 1995/03/01. . 7721471 10.1016/0020-1383(95)92180-i

[5] Young JW, Burgess AR, Brumback RJ, Poka A. Pelvic fractures: value of plain radiography in early assessment and management. Radiology. 1986;160(2):445–51. Epub 1986/08/01. doi: 10.1148/radiology.160.2.3726125 . 3726125 10.1148/radiology.160.2.3726125

[6] Bohme J, Lingslebe U, Steinke H, Werner M, Slowik V, Josten C, et al The extent of ligament injury and its influence on pelvic stability following type II anteroposterior compression pelvic injuries—A computer study to gain insight into open book trauma. Journal of orthopaedic research: official publication of the Orthopaedic Research Society. 2014;32(7):873–9. Epub 2014/03/26. doi: 10.1002/jor.22618 . 24664964 10.1002/jor.22618

[7] Doro CJ, Forward DP, Kim H, Nascone JW, Sciadini MF, Hsieh AH, et al Does 2.5 cm of symphyseal widening differentiate anteroposterior compression I from anteroposterior compression II pelvic ring injuries? Journal of orthopaedic trauma. 2010;24(10):610–5. Epub 2010/09/28. doi: 10.1097/BOT.0b013e3181cff42c . 20871248 10.1097/BOT.0b013e3181cff42c

[8] Steinke H, Hammer N, Lingslebe U, Hoch A, Klink T, Bohme J. Ligament-induced sacral fractures of the pelvis are possible. Clinical anatomy (New York, NY). 2014;27(5):770–7. Epub 2014/01/24. doi: 10.1002/ca.22312 . 24452928 10.1002/ca.22312

[9] van den Bosch EW, van Zwienen CM, Hoek van Dijke GA, Snijders CJ, van Vugt AB. Sacroiliac screw fixation for tile B fractures. The Journal of trauma. 2003;55(5):962–5. Epub 2003/11/11. doi: 10.1097/01.TA.0000047899.36102.80 . 14608174 10.1097/01.TA.0000047899.36102.80

[10] Gorczyca JT, Varga E, Woodside T, Hearn T, Powell J, Tile M. The strength of iliosacral lag screws and transiliac bars in the fixation of vertically unstable pelvic injuries with sacral fractures. Injury. 1996;27(8):561–4. Epub 1996/10/01. . 8994561 10.1016/s0020-1383(96)00078-2

[11] Agarwal Y, Doebele S, Windolf M, Shiozawa T, Gueorguiev B, Stuby FM. Two-leg alternate loading model—a different approach to biomechanical investigations of fixation methods of the injured pelvic ring with focus on the pubic symphysis. Journal of biomechanics. 2014;47(2):380–6. Epub 2013/12/03. doi: 10.1016/j.jbiomech.2013.11.008 . 24290178 10.1016/j.jbiomech.2013.11.008

[12] Stuby FM, Doebele S, Agarwal Y, Windolf M, Gueorguiev B, Ochs BG. Influence of flexible fixation for open book injury after pelvic trauma—a biomechanical study. Clin Biomech (Bristol, Avon). 2014;29(6):657–63. Epub 2014/05/24. doi: 10.1016/j.clinbiomech.2014.04.010 . 24852652 10.1016/j.clinbiomech.2014.04.010

[13] MacAvoy MC, McClellan RT, Goodman SB, Chien CR, Allen WA, van der Meulen MC. Stability of open-book pelvic fractures using a new biomechanical model of single-limb stance. Journal of orthopaedic trauma. 1997;11(8):590–3. Epub 1998/01/07. . 9415866 10.1097/00005131-199711000-00008

[14] Walheim G, Olerud S, Ribbe T. Mobility of the pubic symphysis. Measurements by an electromechanical method. Acta orthopaedica Scandinavica. 1984;55(2):203–8. Epub 1984/04/01. . 6545936 10.3109/17453678408992338

[15] Walheim GG. Stabilization of the pelvis with the Hoffmann frame. An aid in diagnosing pelvic instability. Acta orthopaedica Scandinavica. 1984;55(3):319–24. Epub 1984/06/01. . 6741483 10.3109/17453678408992365

[16] Walheim GG, Olerud S, Ribbe T. Motion of the pubic symphysis in pelvic instability. Scandinavian journal of rehabilitation medicine. 1984;16(4):163–9. Epub 1984/01/01. . 6397853

[17] Panjabi MM, Krag MH, Goel VK. A technique for measurement and description of three-dimensional six degree-of-freedom motion of a body joint with an application to the human spine. Journal of biomechanics. 1981;14(7):447–60. Epub 1981/01/01. . 7276006 10.1016/0021-9290(81)90095-6

[18] Panjabi MM, White AA 3rd, Brand RA. A note on defining body parts configurations. Journal of biomechanics. 1974;7(4):385–7. Epub 1974/08/01. . 4411698 10.1016/0021-9290(74)90034-7

[19] Genant HK, Engelke K, Fuerst T, Gluer CC, Grampp S, Harris ST, et al Noninvasive assessment of bone mineral and structure: state of the art. Journal of bone and mineral research: the official journal of the American Society for Bone and Mineral Research. 1996;11(6):707–30. Epub 1996/06/01. doi: 10.1002/jbmr.5650110602 . 8725168 10.1002/jbmr.5650110602

[20] Kim WY, Hearn TC, Seleem O, Mahalingam E, Stephen D, Tile M. Effect of pin location on stability of pelvic external fixation. Clinical orthopaedics and related research. 1999;(361):237–44. Epub 1999/04/23. . 10212618 10.1097/00003086-199904000-00030

[21] Simonian PT, Routt ML Jr., Harrington RM, Tencer AF. Internal fixation of the unstable anterior pelvic ring: a biomechanical comparison of standard plating techniques and the retrograde medullary superior pubic ramus screw. Journal of orthopaedic trauma. 1994;8(6):476–82. Epub 1994/12/01. . 7869161

[22] Vleeming A. The sacro-iliac joint: a clinical-anatomical, biomechanical and radiological study 1990.

[23] Berber O, Amis AA, Day AC. Biomechanical testing of a concept of posterior pelvic reconstruction in rotationally and vertically unstable fractures. The Journal of bone and joint surgery British volume. 2011;93(2):237–44. Epub 2011/02/02. doi: 10.1302/0301-620X.93B2.24567 . 21282765 10.1302/0301-620X.93B2.24567

[24] Kraus E, Schlickewei W, Cordey J, Wahl D, Kuner EH, Perren SM. [Method for measuring the comparative stability of osteosynthesis in the dorsal pelvic ring]. Unfallchirurgie. 1998;24(1):25–31. Epub 1998/05/09. . 9541981

[25] Pohlemann T, Angst M, Schneider E, Ganz R, Tscherne H. Fixation of transforaminal sacrum fractures: a biomechanical study. Journal of orthopaedic trauma. 1993;7(2):107–17. Epub 1993/01/01. . 8459294 10.1097/00005131-199304000-00002

[26] Alton TB, Gee AO. Classifications in brief: young and burgess classification of pelvic ring injuries. Clinical orthopaedics and related research. 2014;472(8):2338–42. Epub 2014/05/29. doi: 10.1007/s11999-014-3693-8 . 24867452 10.1007/s11999-014-3693-8 PMC4079881

[27] Abdelfattah A, Moed BR. Ligamentous contributions to pelvic stability in a rotationally unstable open-book injury: a cadaver study. Injury. 2014;45(10):1599–603. Epub 2014/06/19. doi: 10.1016/j.injury.2014.05.026 . 24938676 10.1016/j.injury.2014.05.026

[28] Bohme J, Steinke H, Huelse R, Hammer N, Klink T, Slowik V, et al [Complex ligament instabilities after "open book"-fractures of the pelvic ring—finite element computer simulation and crack simulation]. Zeitschrift fur Orthopadie und Unfallchirurgie. 2011;149(1):83–9. Epub 2010/11/17. . 21080314 10.1055/s-0030-1250471

[29] Gary JL, Mulligan M, Banagan K, Sciadini MF, Nascone JW, O'Toole R V. Magnetic resonance imaging for the evaluation of ligamentous injury in the pelvis: a prospective case-controlled study. Journal of orthopaedic trauma. 2014;28(1):41–7. Epub 2013/05/18. doi: 10.1097/BOT.0b013e318299ce1b . 23681412 10.1097/BOT.0b013e318299ce1b

[30] Acklin YP, Zderic I, Buschbaum J, Varga P, Inzana JA, Grechenig S, et al Biomechanical comparison of plate and screw fixation in anterior pelvic ring fractures with low bone mineral density. Injury. 2016;47(7):1456–60. Epub 2016/05/02. doi: 10.1016/j.injury.2016.04.013 . 27131409 10.1016/j.injury.2016.04.013

[31] Grechenig S, Gansslen A, Gueorguiev B, Berner A, Muller M, Nerlich M, et al PMMA-augmented SI screw: a biomechanical analysis of stiffness and pull-out force in a matched paired human cadaveric model. Injury. 2015;46 Suppl 4:S125–8. Epub 2015/11/07. doi: 10.1016/S0020-1383(15)30031-0 . 26542858 10.1016/S0020-1383(15)30031-0

[32] Gruneweller N, Raschke MJ, Zderic I, Widmer D, Wahnert D, Gueorguiev B, et al Biomechanical comparison of augmented versus non-augmented sacroiliac screws in a novel hemi-pelvis test model. Journal of orthopaedic research: official publication of the Orthopaedic Research Society. 2016 Epub 2016/08/27. doi: 10.1002/jor.23401 . 27564231 10.1002/jor.23401

[33] Sagi HC, Papp S. Comparative radiographic and clinical outcome of two-hole and multi-hole symphyseal plating. Journal of orthopaedic trauma. 2008;22(6):373–8. Epub 2008/07/03. doi: 10.1097/BOT.0b013e31817e49ee . 18594300 10.1097/BOT.0b013e31817e49ee

[34] Godinsky RJ, Vrabec GA, Guseila LM, Filipkowski DE, Elias JJ. Biomechanical comparison of locked versus non-locked symphyseal plating of unstable pelvic ring injuries. European journal of trauma and emergency surgery: official publication of the European Trauma Society. 2016 Epub 2016/04/17. doi: 10.1007/s00068-016-0661-x . 27084539 10.1007/s00068-016-0661-x

[35] Simonian PT, Routt ML Jr., Harrington RM, Mayo KA, Tencer AF. Biomechanical simulation of the anteroposterior compression injury of the pelvis. An understanding of instability and fixation. Clinical orthopaedics and related research. 1994;(309):245–56. Epub 1994/12/01. . 7994968

